# Multimodal imaging-based targeting approach for network-level brain stimulation

**DOI:** 10.3389/fnins.2026.1803897

**Published:** 2026-05-29

**Authors:** Alireza Shahbabaie, Mohamed Abdelmotaleb, Harun Kocataş, Filip Niemann, Daria Antonenko, Agnes Flöel, Marcus Meinzer

**Affiliations:** 1 Department of Neurology, University Medicine Greifswald, Greifswald, Germany; 2 German Center for Neurodegenerative Diseases (DZNE Site Greifswald), Greifswald, Germany

**Keywords:** concurrent tDCS-fMRI, focal tDCS, functional connectivity, memory and language learning, multimodal magnetic resonance imaging, network-level brain stimulation, non-invasive brain stimulation (NIBS)

## Abstract

**Introduction:**

Neural network effects of transcranial direct current stimulation (tDCS) are poorly understood. Here, we introduce a prospective, empirically informed, multimodal functional magnetic resonance imaging (fMRI) framework for guiding target selection and hypothesis-based analysis in future focal tDCS-fMRI studies.

**Methods:**

We illustrate our approach by using data of 37 healthy individuals (19 females; mean age ± SD = 25.8 ± 5.9) recruited from two tDCS-fMRI studies that were acquired at the same scanner and with placebo-tDCS. Participants completed two resting-state (RS) sessions and two task-fMRI sessions (object-location memory, OLM, or associative picture-pseudoword learning, APPL, experiments). Seed-based RS analysis identified functional networks originating from target regions for focal tDCS (right occipito-temporal cortex, rOTC; left ventral IFG, lvIFG) and established their test-retest reliability (TRR), using intraclass correlation coefficients (ICC). Dice coefficients quantified overlap between seeded RS networks and task-evoked activity to identify task-active regions potentially affected by downstream network effects from the target regions.

**Results:**

Seed-based analyses identified highly reliable ventral visual-limbic (rOTC) and language-related networks (lvIFG), with 72-77% of voxels showing good-to-excellent TRR (ICC ≥ 0.75). Only a subset of network voxels identified by the RS analyses overlapped with activity elicited by the experimental paradigms (ranging from 7.5-55%), with larger correspondence for the OLM (Dice: 0.249-0.349; APPL 0.065-0.106). Therefore, the degree of potential tDCS network effects varied substantially depending on the target region, the extent of its functional network and task-specific activity patterns. Degree of correspondence was further mediated by the selected contrasts-of-interest in the task-based analyses, with more conservative control conditions resulting in reduced overlap.

**Conclusion:**

In sum, we established a principled multimodal fMRI framework bridging a critical gap in neuromodulation research. By integrating reliable intrinsic connectivity maps with task-evoked activity patterns, we provide a method to prospectively identify network-level targets for focal brain stimulation and generate hypotheses for tDCS-fMRI analyses. This approach shifts the rationale from stimulating isolated brain regions to strategically targeting key nodes within a predefined functional pathway.

## 1 Introduction

Transcranial direct current stimulation (tDCS) applies weak electrical currents via scalp-attached electrodes to modulate excitability and plasticity in the human brain (Stagg et al., 2018) and beneficial effects of tDCS on motor and cognitive functions have been demonstrated in healthy and clinical populations (Kang et al., 2024; Lv et al., 2024; Narmashiri and Akbari, 2025). However, while the basic neurophysiological mechanisms of tDCS are well understood (Cirillo et al., 2017), its effects on the complex neural networks underlying human brain functions requires further investigation. Although carefully designed behavioral studies can provide evidence for strong brain-behavior relationships (e.g., Gbadeyan et al., 2016a; Martin et al., 2020), they are limited to specific target regions. In addition, computational models can be used to simulate the distribution and intensity of the induced current, to investigate which brain regions are potentially affected by tDCS (Hunold et al., 2023). Therefore, combining tDCS with functional neuroimaging is essential to reveal its effects on large-scale functional brain networks and their relationship to behavioral modulation.

Functional magnetic resonance imaging (fMRI) offers high spatial and sufficient temporal resolution to study tDCS effects at the whole brain level (Esmaeilpour et al., 2020). By administering tDCS concurrently with task-based or resting-state (RS) fMRI, it is possible to investigate immediate stimulation effects on brain function. Furthermore, structural data acquired in the same session can be used for individualized current modeling and to verify accurate electrode placement (Meinzer et al., 2024; Niemann et al., 2024a). Using this approach, several studies have demonstrated that tDCS can modulate local brain activity at the stimulation site, activity in remote areas and also large-scale functional network organization (for a review, see Ghobadi-Azbari et al., 2021). However, the vast majority of other concurrent tDCS-fMRI studies have employed conventional montages using relatively large electrodes (25–100 cm^2^), resulting in widespread current flow between electrodes, potentially affecting multiple brain regions and networks simultaneously (e.g., Saturnino et al., 2019). Consequently, the origin of the observed changes in brain function (e.g., neural network modulation vs. current spread) remained difficult to ascertain.

More recently developed focal tDCS protocols use smaller electrodes and concentric arrangement of cathodes around a center anode, to allow more precise current delivery compared to conventional montages (e.g., Ostrowski et al., 2022; Niemann et al., 2024a). This improved focality raises the possibility for network-specific neural modulation, but focal tDCS-fMRI studies are scarce (e.g., Gbadeyan et al., 2016b; Lefebvre et al., 2019; Lim et al., 2024; Müller et al., 2023) and there is currently no established framework for testing hypotheses regarding network-specific neural modulation (Esmaeilpour et al., 2020; Opitz et al., 2018). To address this knowledge gap, the present study aimed to develop a predictive, empirically informed framework that integrates RS networks and task-evoked activity to prospectively identify potential substrates for network-level modulation in focal tDCS-fMRI studies (Note: this study was not designed to reveal the mechanisms of active tDCS). To illustrate our approach, we used data obtained from the preparatory stage of a large-scale, multicenter tDCS-fMRI study that only administered sham tDCS (Research Unit 5,429, RU; www.memoslap.de/en/home/). Specifically, task- and RS fMRI data from two RU projects that assessed neural correlates of object-location memory (OLM) and associative picture-pseudoword learning (APPL) tasks were used. These projects were selected as examples for testing our framework for two main reasons: First, OLM and APPL engage well-characterized and regionally distinct neural networks and allowed selection of functionally relevant and distant target regions for tDCS (e.g., Gillis et al., 2016; Grill-Spector et al., 2001; Rodríguez-Fornells et al., 2009; Tagarelli et al., 2019). In particular, the right occipito-temporal cortex (rOTC) was identified as a critical hub for OLM, and the left ventral inferior frontal gyrus (lvIFG) for APPL, in the respective tasks in the original studies (Abdelmotaleb et al., 2025; Kocataş et al., 2025). Second, data were acquired with identical acquisition parameters at the same scanner and the structure of the experimental tasks was identical, thereby facilitating comparison of study outcomes (please see below for details). In a first step, we employed seed-based RS data analysis to identify the functional networks associated with potential target regions for tDCS in the respective projects (i.e., rOTC, lvIFG) and also established their test–retest reliability (TRR) using intraclass correlation coefficients (ICC). Second, we identified task-based activity patterns elicited by the respective learning tasks. Third, we assessed the overlap between the RS functional networks (from step one) and the task-active regions (from step two) to identify which task-based regions could potentially serve as substrates for network-level propagation of tDCS effects using focal concurrent sham tDCS-fMRI experiments. Hence, we aimed to develop a predictive, empirically informed, multimodal network-based framework (i.e., combining RS connectivity and task-evoked activation within a single analytical pipeline), suited for guiding target selection of future focal tDCS-fMRI studies and for guiding hypothesis-based data analysis by identifying where network-level effects are most likely to occur.

## 2 Materials and methods

The present study was conducted in the context of an ongoing multicenter crossover tDCS-fMRI study, aimed at investigating the behavioral and neural effects of tDCS on learning and memory across functional domains in healthy individuals.^1^ The initial phase of the overall project involved repeated assessment of the different cognitive or motor paradigms used in eight experimental Research Unit (RU) projects (a) to identify potential target regions for active tDCS administration in later project stages and (b) to assess TRR of behavioral and neural outcomes in a repeated measures design. This phase of the project only involved sham tDCS.

To illustrate our methodological approach, we used data from this initial phase obtained from two projects that were acquired on the same scanner using identical acquisition parameters. The experimental setup (e.g., tasks, electrode configurations and neuronavigated placement, Niemann et al., 2024b), was identical to that of subsequently planned active focal tDCS arms within the larger project, and included sham tDCS in all sessions (see section 2.1.4 for details). This approach ensured that the present data can serve as a direct comparator for future studies within the consortium. The study was pre-registered (Open Science Framework; https://osf.io/t37u2).

### 2.1 Participants

A total of 37 healthy young to middle-aged participants (19 female; mean age ± SD = 25.8 ± 5.9, range: 19–42) were included in the study. Participants were subsequently assigned to complete either the OLM task (*n* = 18; 8 females; mean age ± SD = 25.5 ± 5.5) or the APPL task (*n* = 19; 11 females; mean age ± SD = 26.1 ± 6.3). Interested participants were screened tDCS-fMRI eligibility via telephone and completed a comprehensive baseline assessment to ensure normal cognitive functions, followed by four combined tDCS-fMRI sessions (two RS and two task-based fMRI sessions). All participants were right-handed based on the Edinburgh Handedness Inventory (Oldfield, 1971) native German speakers, and did not report any past or current neurological or psychiatric diseases. All experimental procedures were approved by the medical ethics committee of the University Medicine Greifswald. Prior to their study inclusion, all participants provided informed consent and received monetary compensation upon study completion.

### 2.2 Procedure

The four fMRI sessions were conducted at least 1 week apart. RS data acquisition comprised two consecutive 10-min runs per session. Participants were instructed to keep their eyes open while fixating on a central cross-hair. Afterwards, participants were assigned to the task-based fMRI groups and completed either the OLM (*n* = 18) or APPL (*n* = 19) tasks.

### 2.3 Cognitive task-based imaging

Presentation of the two task-related paradigm (OLM, APPL) used Presentation® software (Version 20.1, Neurobehavioral Systems, Inc., Berkeley, CA, USA) and an MRI-compatible back-projection system. Behavioral responses were recorded using MRI-compatible response grips (NordicNeuroLab, Norway; www.nordicneurolab.com). In order to minimize potential learning effects, two parallel versions of each task were administered in a counterbalanced order across sessions. Control conditions followed the same structure, but did not involve learning (see below for details).

#### 2.3.1 Object-location memory (OLM) task

The OLM task, adapted from prior studies (Antonenko et al., 2018; De Sousa et al., 2020; Flöel et al., 2012; Prehn et al., 2017) and required participants to learn associations between objects (houses) and their spatial locations on a 2-dimensional map. The task employed a block design consisting of four learning stages, integrating both instruction, and feedback-based learning mechanisms. The control condition retained the same structure but was devoid of associative learning, requiring only left/right decisions about houses on the map. Further details on the intra-scanner task design are provided in Abdelmotaleb et al. (2025).

#### 2.3.2 Associative picture-pseudoword learning (APPL) task

The APPL task was adapted from Sliwinska et al. (2017) and involved learning associations between pictures of common objects and pseudowords using an explicit instruction- and reinforcement-based paradigm. The experiment employed a block design, encompassing six APPL blocks and two control task blocks, with 40 trials per block. While the overall structure of the APPL and control tasks was identical, only the APPL condition involved active associative learning. Further details on the intra-scanner task design are provided in Kocataş et al. (2025).

### 2.4 Focal tDCS

Focal sham tDCS was administered with a 3 × 1 tDCS montage using an MRI-compatible stimulator (DC-STIMULATOR MC, NeuroConn GmbH). The sham tDCS protocol involved a 10-s ramp-up to 2 mA, 20 s of active stimulation, and a 10-s ramp-down. The anode was placed over individual target regions (rOTC for OLM, lvIFG for APPL) and surrounded by three equally spaced cathodes (Niemann et al., 2024b). Individualized electrode placement was based on subject-specific electric field simulations derived from structural MRI data (for details see Thielscher et al., 2026). In brief, for each participant, the target region was mapped into individual space, and the center (anodal) electrode position was determined by minimizing the Euclidean distance between the scalp surface and the cortical target, ensuring that the induced electric field was centered over the region of interest. While electrode positions were individualized to account for inter-individual anatomical variability in current flow, the center-surround geometry (i.e., electrode distances) was kept constant across participants to ensure comparable stimulation conditions at the group level. The overall procedure ensured precise targeting and provides a direct comparator for future active tDCS arms within the consortium.

#### 2.4.1 Adverse effect questionnaire

After each of the four scanning sessions, participants completed a questionnaire assessing potential adverse effects (AEs) of tDCS. The questionnaire evaluated six possible AEs: itching, pain, burning, warmth/heat, metallic/iron taste, and fatigue/reduced attention. Each item was rated on a 4-point scale (0 = none, 1 = mild, 2 = moderate, 3 = strong).

### 2.5 MRI acquisition

All data were acquired on a 3 T Siemens MAGNETOM Vida scanner with a 64-channel head–neck coil (Siemens Healthineers, Germany). Functional MRI used a multiband EPI sequence (CMRR, University of Minnesota, www.cmrr.umn.edu/multiband) with the simultaneous multislice (SMS) acceleration for high temporal resolution and whole-brain coverage (Setsompop et al., 2012). Key acquisition parameters included: 2 × 2 × 2 mm^3^ voxels, TR = 1,000 ms, TE = 30 ms, flip angle = 60°, FOV = 220 mm, multiband factor = 6, GRAPPA acceleration = 2, and anterior–posterior phase encoding Parameters: 2 × 2 × 2 mm^3^ voxels, TR = 1,000 ms, TE = 30 ms, flip angle = 60°, FOV = 220 mm, multiband factor = 6, GRAPPA acceleration = 2, anterior–posterior phase encoding. Resting-state sessions comprised two 10-min runs (600 volumes each); task sessions lasted ~30 min (1800 volumes). Field maps were acquired for distortion correction.

High-resolution T1-weighted MPRAGE (0.9 mm isotropic; TR = 2,700 ms, TE = 3.7 ms, TI = 1,090 ms) and T2-weighted images (0.9 mm isotropic; TR = 2,500 ms, TE = 349 ms) were acquired for structural reference and normalization of functional images.

### 2.6 Statistical analysis

#### 2.6.1 Preprocessing

RS-fMRI data preprocessing was conducted using fMRIPrep 24.1.1 (Esteban et al., 2019), implementing a standardized pipeline for reproducible analysis. Fieldmap estimation utilized FSL’s topup based on available EPI references to correct B0 inhomogeneities. For anatomical data, T1-weighted images underwent N4 bias field correction, skull-stripping using ANTs (OASIS30ANTs template), and tissue segmentation via FSL’s FAST. Surface reconstruction was performed with FreeSurfer 7.3.2, incorporating T2-weighted data for improved pial surface estimation. Spatial normalization transformed data to both MNI152NLin6Asym and MNI152NLin6Asym:res-02 standard spaces using ANTs’ nonlinear registration.

Functional preprocessing included motion correction (FSL’s mcflirt), fieldmap-based distortion correction, and boundary-based coregistration to anatomical space (FreeSurfer’s bbregister). Confound regressors comprised framewise displacement (FD > 0.5 mm threshold), the temporal Derivative of root mean square VARiance over voxelS (DVARS), anatomical and temporal CompCor components (50% variance explained), and global signals. All transformations were combined in a single interpolation using cubic B-spline sampling through nitransforms. We visually inspected the automatically generated quality control metrics at each processing stage to verify data quality.

#### 2.6.2 Confound time series removal

We implemented a denoising pipeline following an optimized strategy recently suggested by Wang et al. (2023) in their systematic comparison of resting-state fMRI denoising strategies. The confound time series generated during fMRIPrep preprocessing were used to extract nuisance regressors.

Our denoising model incorporated 27 regressors, comprising a comprehensive set of 24 motion parameters (Friston et al., 1994; Satterthwaite et al., 2013) to capture both linear and nonlinear motion artifacts while maintaining orthogonality between regressors, global signal (GS), and mean signals from white matter (WM) and cerebrospinal fluid (CSF).

We automated this workflow through a custom MATLAB script (R2025a) utilizing SPM12 (version 7,771) and the CONN toolbox (v22.2407, RRID: SCR_009550) with its batch processing functionality [available at (https://github.com/ShahAliR/memoslap-denoising-pipeline)] to enhance quality assurance and enable seed-based analyses within the same computational framework.

#### 2.6.3 Seed-based functional connectivity

Seed regions for the RS analysis were determined by the envisaged target regions for focal tDCS, based on their established relevance for the respective learning tasks (rOTC for OLM: Gillis et al., 2016; Rolls et al., 2024; lvIFG for APPL: Perceval et al., 2020; Meinzer et al., 2012; Riemann et al., 2024) studies. Two spherical seed regions (6 mm radius) were created and centered on the MNI coordinates 47, −64, −11 (rOTC) and −47, 20, 2 (lvIFG). Binary masks for these seeds were created using Nilearn (Abraham et al., 2014) and imported into the CONN toolbox for seed-based connectivity analysis.

First-level seed-to-voxel connectivity maps were calculated by correlating the mean BOLD time series of each seed region with every other voxel in the brain, separately for each session and subject. These correlation coefficients were Fisher z-transformed to improve normality for group-level analysis. At the group level, one-sample *t*-tests were conducted across all 37 participants, including both sessions for each subject. This analysis tested whether the average Fisher z-transformed connectivity, aggregated over both sessions and all subjects, differed significantly from zero. Statistical significance was determined by applying a cluster-level family-wise error (FWE) correction at *p* < 0.05, using an initial voxel-wise threshold of *p* < 0.001. For each significant cluster, the peak t-value, MNI coordinates, and cluster size (k) are reported.

#### 2.6.4 Reliability assessment

Test–retest reliability of lvIFG and rOTC functional connectivity maps was assessed using voxel-wise intraclass correlation coefficients (ICC) via Pingouin (Vallat, 2018). We computed ICC(3,k) maps using a two-way mixed-effects model for absolute agreement across sessions.

Significant clusters from seed-based connectivity analyses (FWE-corrected, p < 0.05) served as binary masks to extract ICC values within each functional network. Voxels were classified into reliability tiers (Koo and Li, 2016): poor (ICC < 0.50), moderate (0.50–0.75), good (0.75–0.90), and excellent (ICC ≥ 0.90).

#### 2.6.5 Task-based fMRI analysis

Task-based fMRI data for the OLM and APPL paradigms were analyzed separately using SPM12 (v7771) in MATLAB R2022a. Structural T1-weighted images were skull-stripped, bias-corrected, and normalized to MNI space. Functional BOLD images were preprocessed with realignment, unwarping, slice-time correction (TR = 1 s, 72 slices), co-registration to the structural T1, normalization using the resulting deformation fields, and spatial smoothing with a 6-mm FWHM Gaussian kernel. First-level general linear models (GLMs) were constructed for each participant, incorporating data from both fMRI sessions into a single design matrix. The models included regressors for trial onsets and durations of learning and control conditions across four stages, convolved with the canonical hemodynamic response function. Six motion parameters and framewise displacement were included as nuisance regressors. Subject-specific contrast maps for the primary effect (Learning > Implicit baseline, and Learning > Control) were generated and subsequently entered into second-level one-sample t-tests. Group-level statistical maps were thresholded at *p* < 0.001 (voxel-level) and cluster-corrected for family-wise error (FWE) at *p* < 0.05. The resulting activation maps were overlaid on an MNI template, anatomically labeled using the Harvard–Oxford atlas, and visualized with MRIcroGL. More details of the task-based fMRI analyses for the OLM and APPL paradigms are provided in Abdelmotaleb et al. (2025) and Kocataş et al. (2025), respectively.

#### 2.6.6 Spatial overlap analysis

An identical analytical pipeline was applied to the both functional networks, wherein resting-state connectivity maps from the rOTC seed were compared to OLM task activation, and those from the lvIFG seed were compared to APPL task activation. We further considered effects of specific analytical choices for task-based activity analyses by investigating both the complex (learning > control task) and simple (learning > implicit baseline) contrasts. Spatial correspondence was quantified using the Dice similarity coefficient and voxel-wise Pearson correlation of t-values within a gray-matter mask. Analyses were conducted in Python (v3.x) using NiBabel and Nilearn. Code availability: https://github.com/ShahAliR/NetworkOverlap-Task-RS-fMRI.

#### 2.6.7 Adverse effects assessment

All statistical analyses were conducted in R (version 4.3.3; R Core Team, 2024) within the RStudio environment (Version 2026.04.0+526; Posit team, 2026). To examine the effects of stimulation montage (lvIFG vs. rOTC) and session on AEs intensity, we employed cumulative link mixed models (CLMMs). This approach was selected to appropriately model the ordinal nature of the AEs intensity ratings (0–3) while accounting for the study’s repeated-measures design by incorporating a random effect for participant.

## 3 Results

### 3.1 Resting-state functional connectivity

Whole-brain functional connectivity analyses revealed distinct large-scale networks for each seed. The rOTC seed engaged a ventral visual-limbic network, associated with integration of object recognition with contextual memory processing (Gillis et al., 2016; Kravitz et al., 2013; Rolls et al., 2024). The lvIFG seed mapped onto a left-lateralized language-semantic control network supporting lexical, phonological and conceptual integration (Fedorenko et al., 2012; Tagarelli et al., 2019). All reported clusters survived rigorous cluster-level FWE correction (*p* < 0.05).

#### 3.1.1 Seed-based functional connectivity of the rOTC

**Figure 1:**
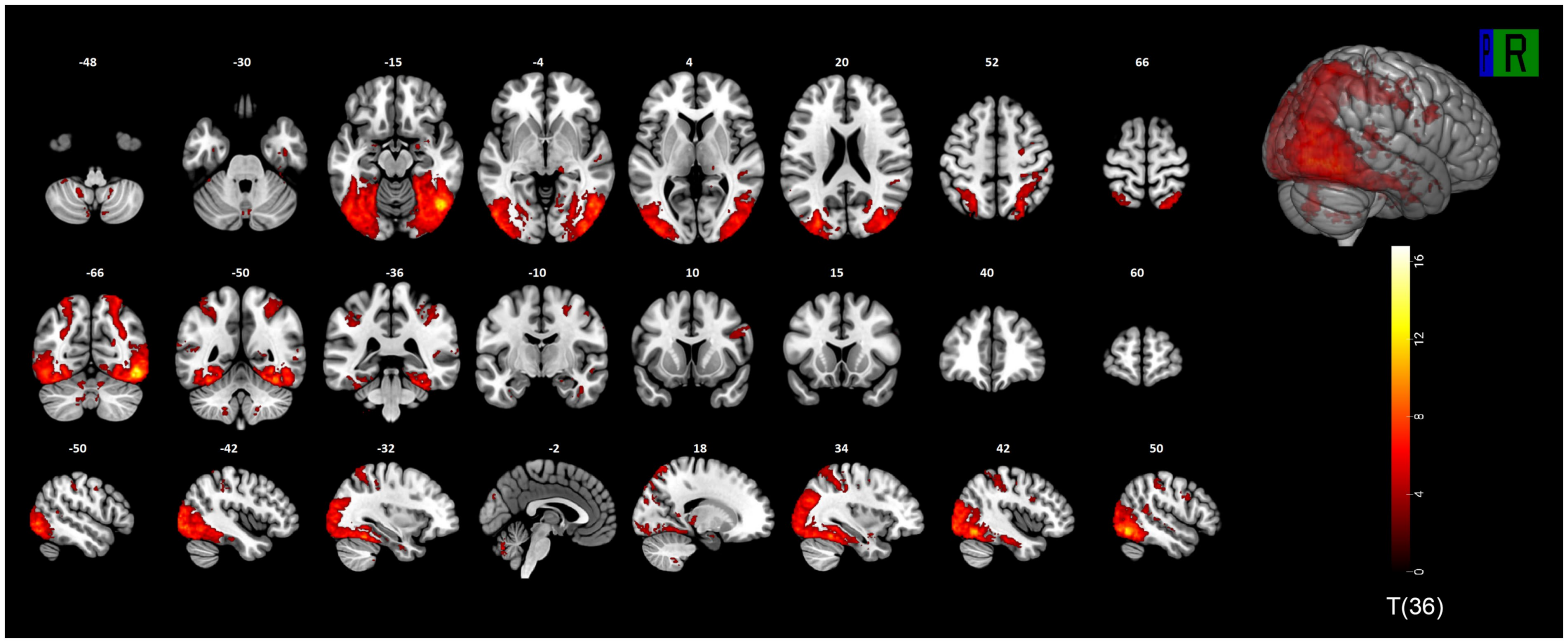
Functional connectivity of the right occipito-temporal cortex (rOTC). Whole-brain positive functional connectivity map for the rOTC seed, overlaid on the MNI152 high-resolution T1-weighted template. Connectivity strength is represented by a graded *T*-statistic scale from dark red (lower *T*-values) to bright yellow (highest *T*-values). Displayed clusters met voxel-wise significance at *p* < 0.001 and survived cluster-level family-wise error (FWE) correction at *p* < 0.05. Multiple axial, sagittal, and coronal slices are presented to capture the complete spatial distribution of the network.

Using the rOTC as the seed region, whole-brain connectivity analysis revealed a distributed network comprising occipital, temporal, parietal, frontal, subcortical, and cerebellar regions (Supplementary Table 1; Figure 1). The most robust positive connectivity was observed with the right occipital fusiform gyrus (lateral visual stream; peak T = 17.70, cluster size = 8,973 voxels, *p* < 0.000001 FWE-corrected) and the left occipital pole (medial early visual cortex; peak T = 11.19, cluster size = 6,682 voxels, *p* < 0.000001). Connectivity extended to higher-order association areas, including the left supramarginal gyrus (posterior division), bilateral superior temporal gyrus, and temporal fusiform cortex. Subcortical associations included the bilateral amygdala (laterobasal and centromedial subnuclei) and parahippocampal gyrus (anterior and posterior collateral sulcus). Additional significant clusters included the cerebellar Crus I, lateral occipital cortex (superior division), and bilateral precentral and postcentral gyri.

#### 3.1.2 Seed-based functional connectivity of the lvIFG

**Figure 2:**
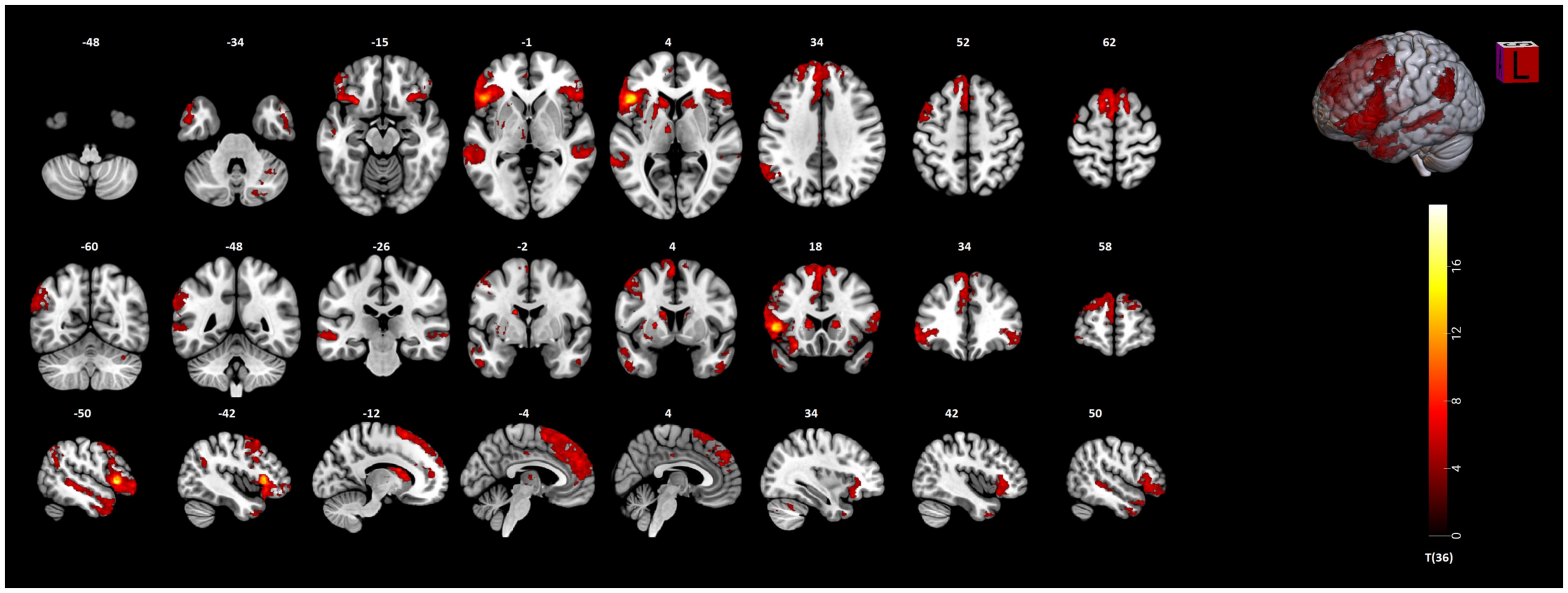
Functional connectivity of the left ventral inferior frontal gyrus (lvIFG). Positive functional connectivity network of the lvIFG seed, displayed using a red-to-yellow *T*-value spectrum on the MNI152 high-resolution T1-weighted template. Dark red regions indicate weaker and bright yellow indicate stronger connectivity. All clusters survived family-wise error (FWE) correction at p < 0.05 (cluster-level), thresholded at voxel-wise *p* < 0.001. Axial, coronal, and sagittal slices are shown to illustrate the full spatial extent of the network.

Seeding the lvIFG, revealed a network predominantly involving temporal, parietal, and cingulo-opercular regions, as well as subcortical and cerebellar structures (Supplementary Table 2; Figure 2). The largest cluster encompassed the left superior temporal gyrus (anterior division; peak T = 20.32, cluster size = 13,145 voxels, *p* < 0.000001) extending into adjacent auditory and language-related cortices. Significant bilateral temporal lobe connectivity included the right temporal pole (peak T = 9.26) and right middle and inferior temporal gyri. Additional strong associations were observed in the left angular gyrus (PGp), bilateral supramarginal gyrus, occipital fusiform gyrus, and precuneus. Subcortical connectivity was identified in the left thalamus (pulvinar), right thalamus (mediodorsal), bilateral caudate, and left hippocampus. Midline connectivity encompassed the anterior and mid-posterior cingulate cortices, as well as the brainstem (midbrain).

### 3.2 Reliability of seed-based functional networks

#### 3.2.1 Reliability of the rOTC functional network

**Figure 3:**
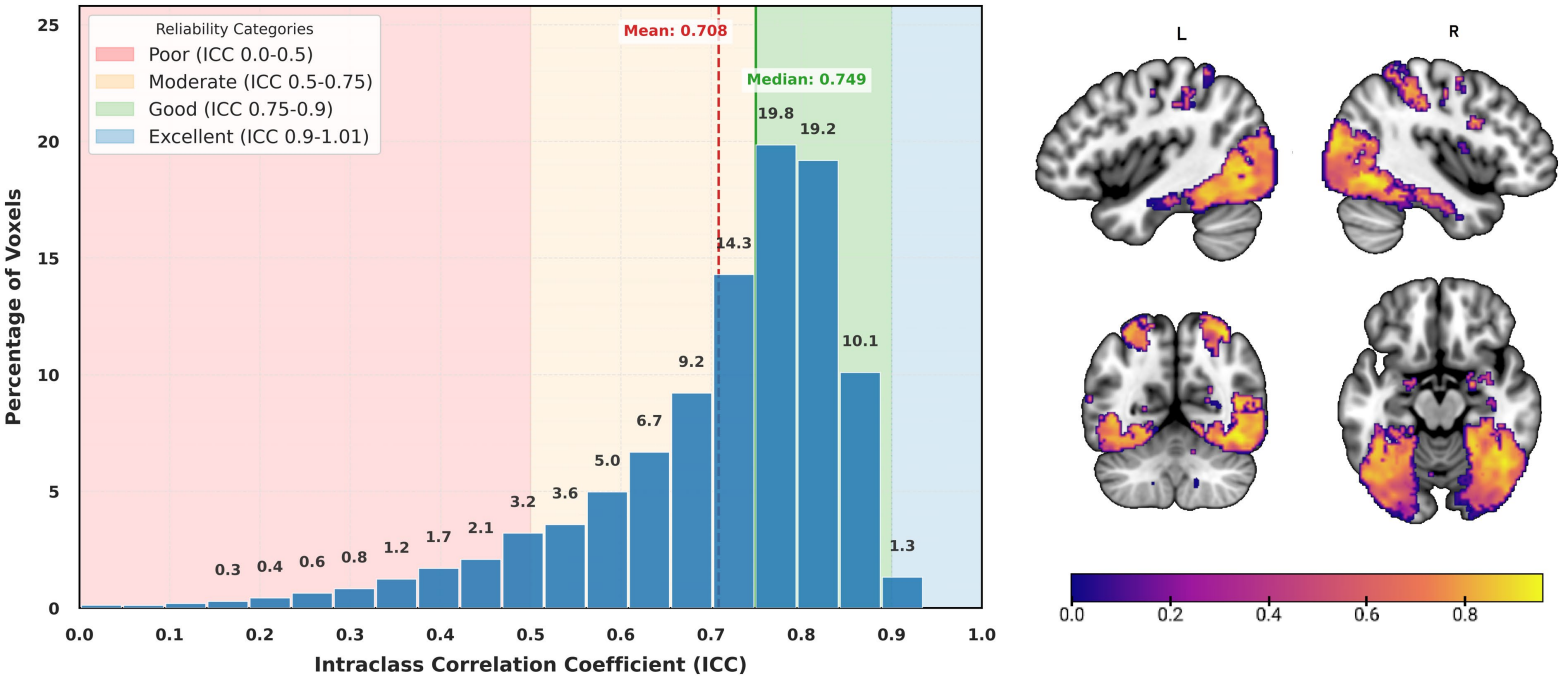
Test–retest reliability within the right occipito-temporal cortex (rOTC) functional network. (Left) Histogram of voxel-wise intraclass correlation coefficient (ICC) values within the rOTC network (*N* = 16,067 voxels). The *x*-axis represents ICC values binned into 20 intervals; the *y*-axis shows the percentage of voxels within each bin. The percentage value atop each column indicates the proportion of voxels within that specific ICC bin. Colored backgrounds denote established reliability tiers: poor (ICC < 0.50), moderate (0.50 ≤ ICC < 0.75), good (0.75 ≤ ICC < 0.90), and excellent (ICC ≥ 0.90). The red dashed vertical line indicates the mean ICC (0.70); the solid green line indicates the median ICC (0.74). (Right) Spatial distribution of ICC values within the rOTC network. Axial, coronal, and sagittal (left and right hemisphere) views are displayed on the MNI152 template. The color bar indicates ICC magnitude, matching the scale of the histogram. Voxels with the highest reliability (ICC > 0.85) are predominantly localized in the fusiform gyrus and lateral occipital cortex.

The rOTC network demonstrated high TRR (Figure 3). The distribution of intraclass correlation coefficient (ICC) values across the 16,067 voxels within the network had a mean of 0.70 (SD = 0.14) and a median of 0.74. The interquartile range (IQR) spanned from 0.64 (25th percentile) to 0.76 (75th percentile).

The majority of voxels within the rOTC network demonstrated good-to-excellent reliability: 72.0% of voxels showed good or excellent reliability (ICC ≥ 0.75), while 25.1% showed moderate reliability (0.50 ≤ ICC < 0.75), and only 2.9% demonstrated poor reliability (ICC < 0.50). The highest reliability values (ICC > 0.85) were predominantly observed in regions most strongly connected to the rOTC seed, including the fusiform gyrus and lateral occipital cortex.

#### 3.2.2 Reliability of the lvIFG functional network

**Figure 4:**
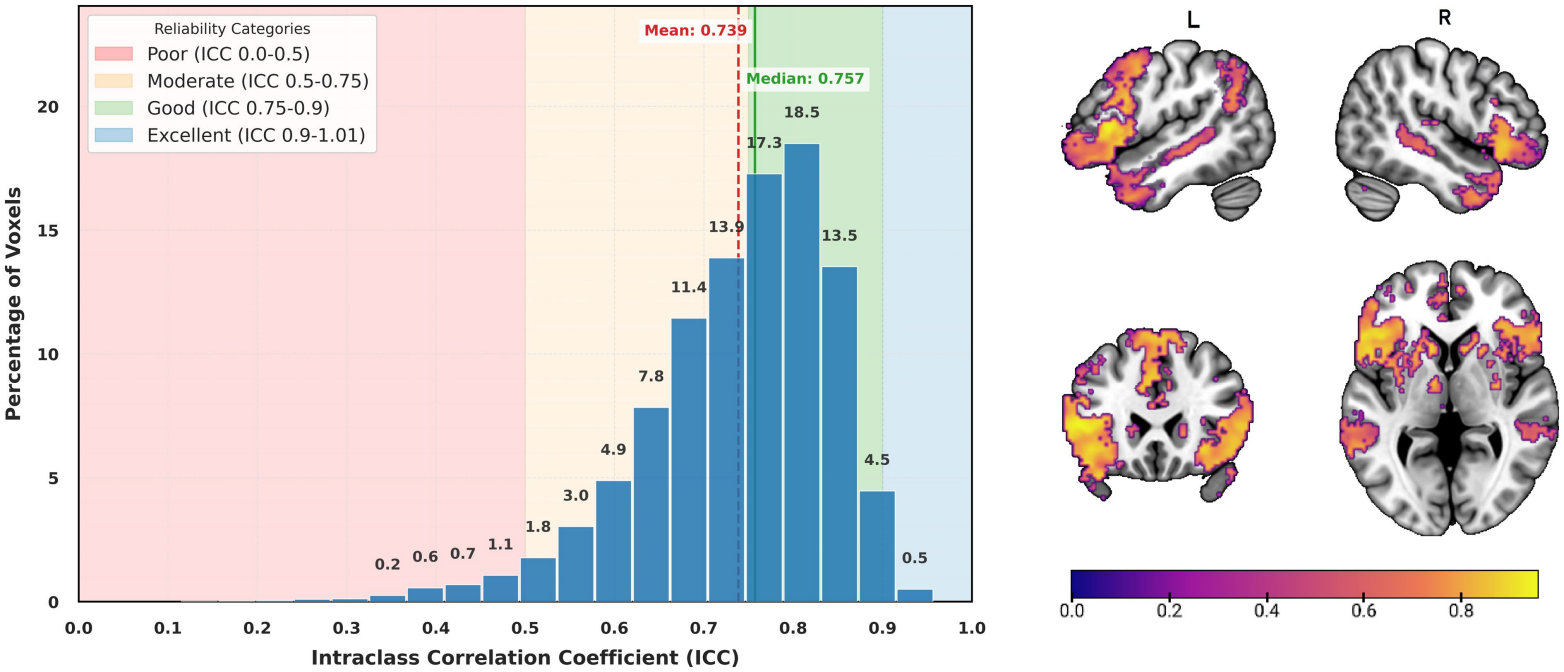
Test–retest reliability within the left ventral inferior frontal gyrus (lvIFG) functional network. (Left) Histogram of voxel-wise intraclass correlation coefficient (ICC) values within the lvIFG network (*N* = 17,216 voxels). The *x*-axis represents ICC values binned into 20 intervals; the *y*-axis shows the percentage of voxels within each bin. The percentage value atop each column indicates the proportion of voxels within that specific ICC bin. Colored backgrounds denote established reliability tiers (Koo and Li, 2016): poor (ICC < 0.50), moderate (0.50 ≤ ICC < 0.75), good (0.75 ≤ ICC < 0.90), and excellent (ICC ≥ 0.90). The red dashed vertical line indicates the mean ICC (0.74); the solid green line indicates the median ICC (0.76). (Right) Spatial distribution of ICC values within the lvIFG network. Axial, coronal, and sagittal (left and right hemisphere) views are displayed on the MNI152 template. The color bar indicates ICC magnitude, matching the scale of the histogram. The network demonstrates a pronounced shift towards higher reliability, with the highest values concentrated in the core regions of the language network.

A similar pattern of high reliability was observed for the lvIFG network (17,216 voxels) (Figure 4). The distribution of ICC values was shifted toward the high end of the reliability spectrum, with a mean of 0.74 (SD = 0.11) and a median of 0.76. The 25th and 75th percentiles were 0.68 and 0.81, respectively.

77.3% of voxels within the lvIFG network demonstrated good or excellent reliability (ICC ≥ 0.75). Moderate reliability was observed in 20.5% of voxels, and only 2.2% showed poor reliability. This confirms that the core functional architecture identified by the lvIFG seed is highly consistent across sessions.

### 3.3 Task-based fMRI

#### 3.3.1 Neural correlates of object-location memory

**Figure 5:**
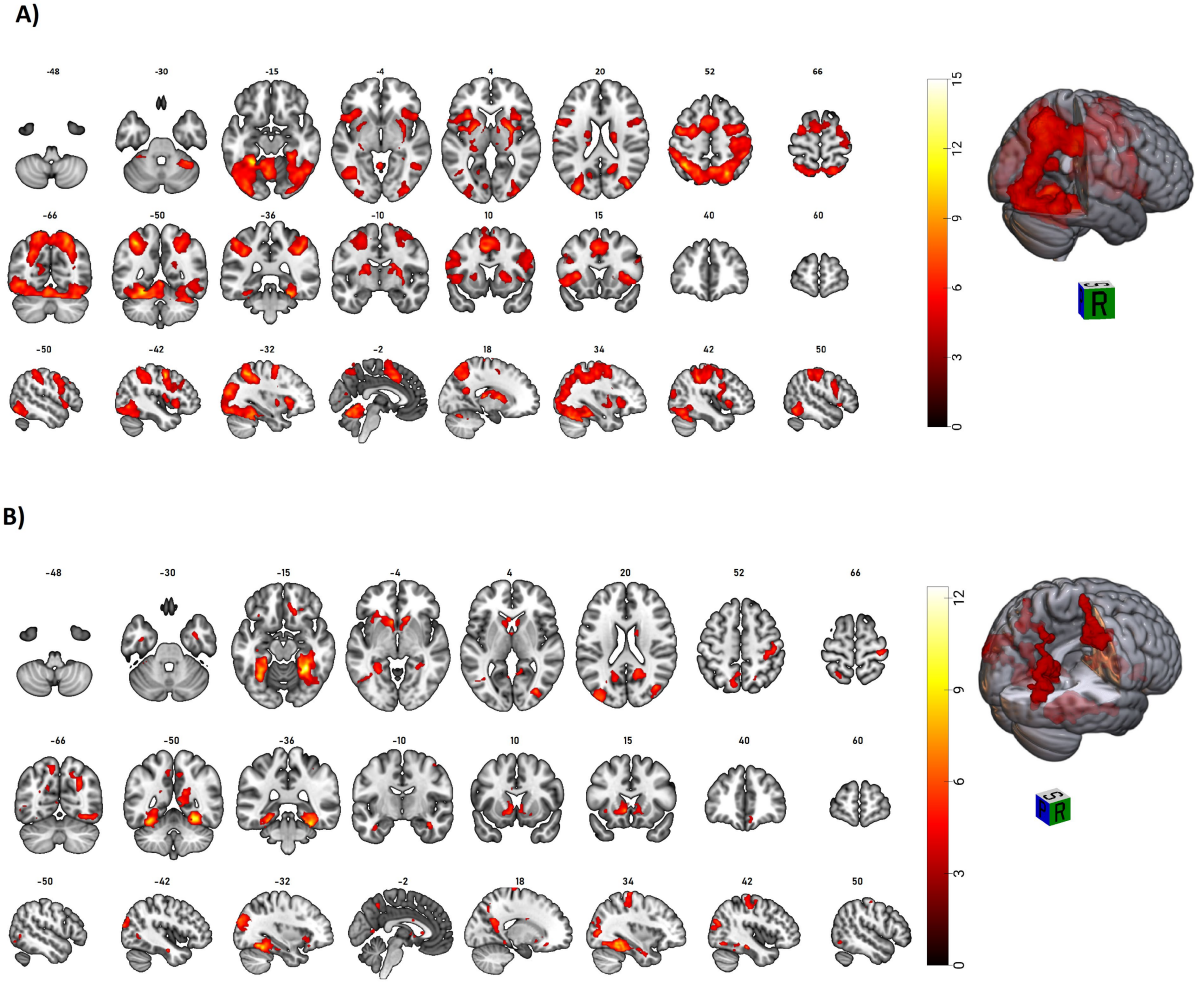
Whole-brain activation maps for the object-location memory (OLM) task. **(A)** Learning > Implicit baseline contrast. **(B)** Learning > Control contrast. All clusters are significant (pFWE < 0.05, cluster-level corrected). The color bar represents the T-score.

Whole-brain analysis revealed robust neural activity during the OLM learning task compared to the implicit baseline (learning > implicit baseline), engaging a widespread network including left medial temporal, fronto-parietal, and bilateral subcortical regions, consistent with the demands of OLM task (see Supplementary Table 3A; Figure 5A). The most extensive activation was a large left-lateralized cluster encompassing the parahippocampal gyrus and superior parietal lobule (SPL).

The direct comparison to the control condition (Learning > Control) highlighted a more specific pattern of activation, with the strongest effects observed bilaterally in the ventral visual stream, including the temporal occipital fusiform cortex and parahippocampal gyrus. Further significant activation was identified in the lateral occipital cortex, right sensorimotor cortex, bilateral precuneus, and subcortical structures (see Supplementary Table 3B; Figure 5B).

#### 3.3.2 Neural correlates of associative picture-pseudoword learning

**Figure 6:**
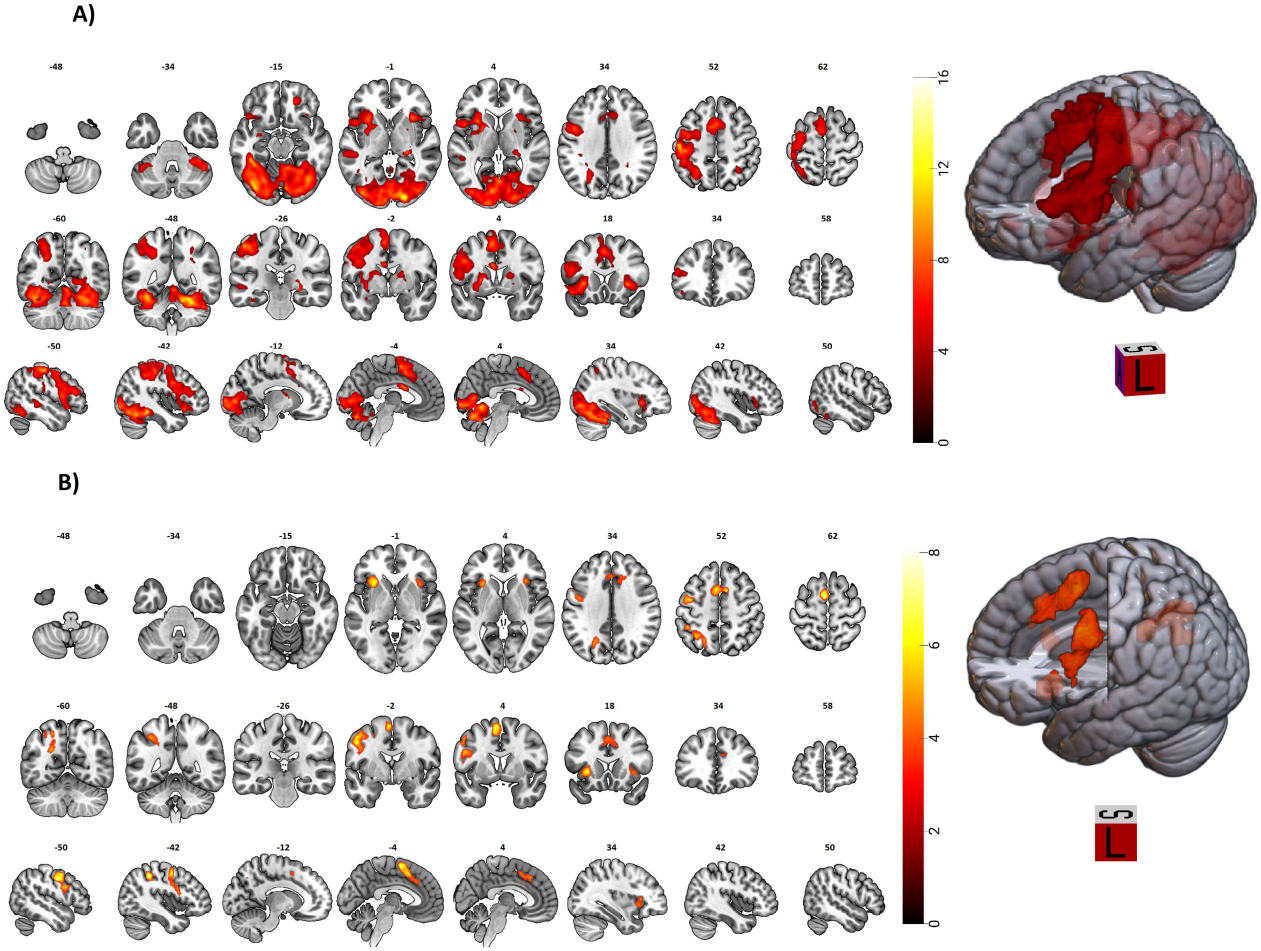
Whole-brain activation maps for the associative picture-pseudoword learning (APPL) task. **(A)** Learning > Implicit baseline contrast. **(B)** Learning > control contrast. All clusters are significant (*p*_FWE_ < 0.05, cluster-level corrected). The color bar represents the *T*-sco*re.*

Whole-brain analysis revealed widespread neural activity during the APPL learning task compared to the implicit baseline (Learning > Implicit Baseline), encompassing lateral occipital, fusiform, Lingual, superior parietal, and insular regions, consistent with the demands of associative novel-word learning (see Supplementary Table 4A; Figure 6A). The most pronounced activations were found in a large bilateral cerebellar cluster, a left-dominant fronto-parietal, and temporal occipital networks.

The direct comparison to the control condition (Learning > Control) identified a more focused set of regions specifically implicated in successful encoding. The strongest effects were found in bilateral cognitive control regions, including the SMA and paracingulate gyrus, the bilateral insular cortex, and the left precentral gyrus. (see Supplementary Table 4B; Figure 6B).

### 3.4 Overlap between intrinsic functional networks and task-evoked activation

#### 3.4.1 Convergence of the intrinsic rOTC network with OLM task

We quantified the spatial correspondence between the intrinsic rOTC network and task-evoked activity during the OLM task. A significant positive spatial correlation was observed for both the OLM > Control contrast (*r* = 0.289, *p* < 0.001) and the OLM > Baseline contrast (*r* = 0.365, *p* < 0.001). This indicates that voxels exhibiting higher task-evoked activation also demonstrated stronger intrinsic functional connectivity with the rOTC seed.

**Figure 7:**
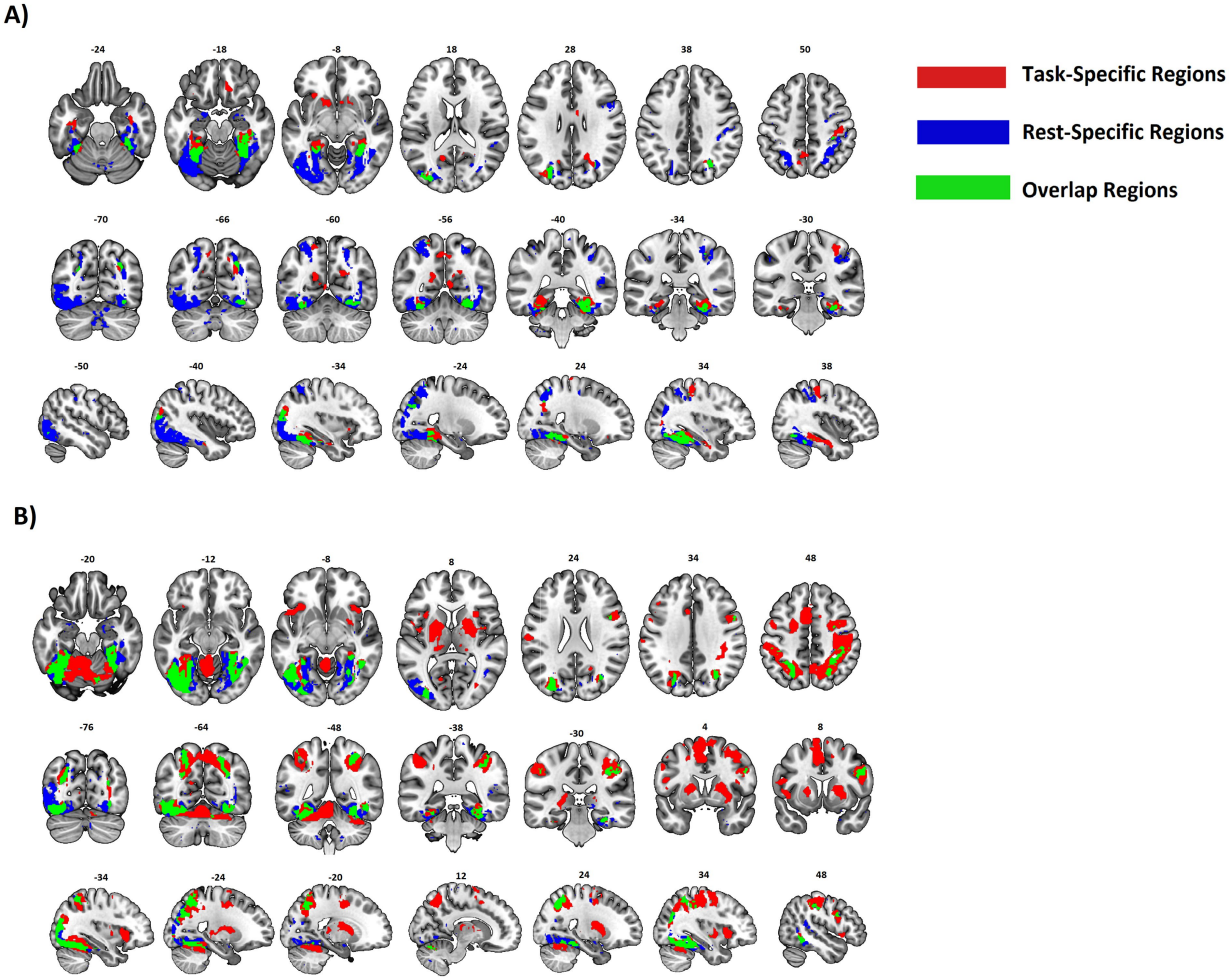
Distinct and shared neural substrates for the OLM task and rOTC network. Multi-planar views show the spatial relationship for the **(A)** Learning > Control and **(B)** Learning > Baseline contrasts. Red: task-specific activation (FWE-corrected). Blue: resting-state connectivity with the rOTC seed (FWE-corrected). Green: overlap, indicating areas both task-activated and functionally connected to the rOTC.

The overlap of supra-threshold voxels was substantial. For the OLM > Control contrast (see Figure 7A), 1,571 out of 4,198 task-activated voxels overlapped with the 8,426-voxel rOTC network (Dice = 0.249).

A notably greater overlap was found for the OLM > Baseline contrast, with 4,639 out of 18,133 task-activated voxels falling within the rOTC network (Dice = 0.349). The spatial distribution of these overlapping regions is visualized in Figure 7B.

#### 3.4.2 Convergence of the intrinsic lvIFG network with APPL task

We quantified the spatial correspondence between the intrinsic lvIFG network and task-evoked activity during the APPL task. A significant positive spatial correlation was observed for both the APPL > Control contrast (*r* = 0.092, *p* < 0.001) and the APPL > Baseline contrast (*r* = 0.036, *p* < 0.001).

**Figure 8:**
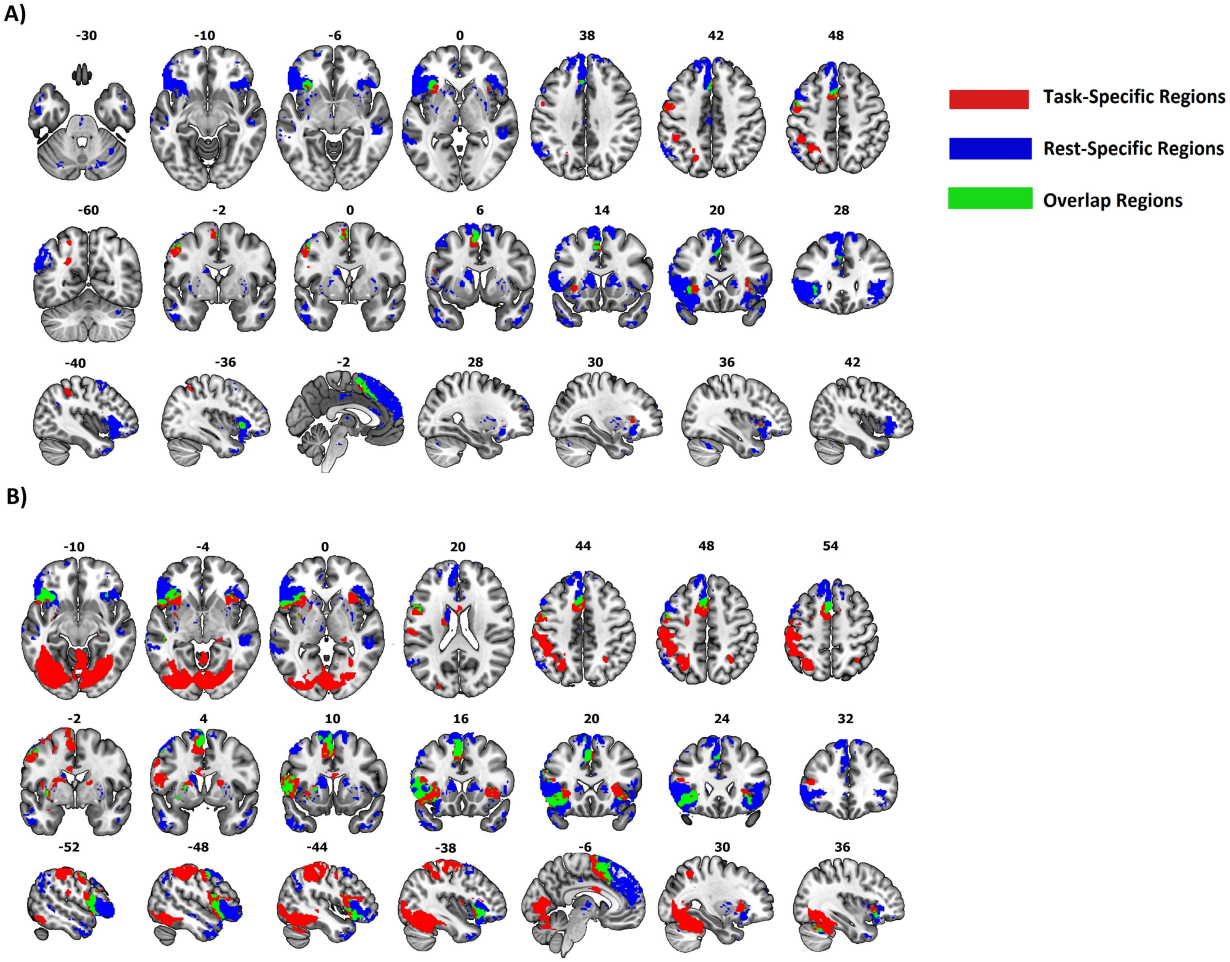
Distinct and shared neural substrates for the APPL task and lvIFG network. Multi-planar views show the spatial relationship for the **(A)** Learning > Control and **(B)** Learning > Baseline contrasts. Red: task-specific activation (FWE-corrected). Blue: resting-state connectivity with the lvIFG seed (FWE-corrected). Green: overlap, indicating areas both task-activated and functionally connected to the lvIFG.

The overlap of supra-threshold voxels was more limited. For the APPL > Control contrast (see Figure 8A), 407 out of 1,382 task-activated overlapped with the 11,082-voxel lvIFG network (Dice = 0.065).

For the APPL > Baseline contrast (see Figure 8B), 1,479 out of 16,853 task-activated voxels fell within the lvIFG network (Dice = 0.106).

### 3.5 Adverse effects

Across the 148 sessions, the reported AEs were classified as follows: fatigue (57.1%), pain (40.1%), burning (38.8%), warmth/heat (36.7%), itching (17.0%), and metallic taste (4.1%). Fatigue and warmth/heat were the most frequently reported mild AEs (28.6% each). Fatigue was also the most frequent moderate (21.1%) and strong (7.5%) AEs. Strong burning and pain were each reported in 1.6% of sessions (*N* = 3 participants, one reported strong AEs in both sessions). Given the complexity of the tDCS-fMRI procedure (and prior neuronavigated electrode placement), a higher level of discomfort was anticipated. Notably, all participants completed the study procedures (see Supplementary Table 5). To examine the effects of stimulation group and session on AEs intensity, we employed CLMMs, which account for the repeated-measures design by incorporating a random effect for participant. For itching, pain, burning, and fatigue, the models converged successfully. Pairwise comparisons revealed no significant differences in AEs intensity between study sessions for either the lvIFG or rOTC group (*p* > 0.05). No significant main effects of group or session, nor group-by-session interaction effects, were identified. Models for metallic taste and warmth/heat failed to converge, likely due to data sparsity resulting from the low frequency of moderate-to-strong severity reports. Consequently, these AEs were analyzed descriptively, indicating low prevalence and high stability across all study sessions.

## 4 Discussion

We describe a cross-modal fMRI-based framework to guide planning of network-specific neuromodulation and empirically guided hypothesis testing in focal tDCS-fMRI studies. We illustrated this approach by using two datasets from the MeMoSLAP consortium, that acquired both RS- and task-based fMRI at the same scanner and with identical acquisition parameters. (1) RS-data delineated the functional networks originating from the envisaged target regions for focal tDCS (rOTC, lvIFG) for two distinct experimental learning paradigms (OLM, APPL). The identified RS networks showed substantial consistency across scanning sessions, with good-to-excellent ICCs in well over 70% of voxels of either network. These findings align with long-term intrinsic connectivity networks reproducibility studies reporting ICC > 0.60 for >70% of connectivity elements (Chou et al., 2012; Guo et al., 2012). This highlights the usability of seed-based RS-fMRI approaches for network-based targeting and hypothesis testing. (2) Subsequently, we determined the spatial overlap of the identified seeded-RS networks with activity elicited by the respective learning paradigms. This step identified regions in the task-active networks that could be affected by focal tDCS, either via direct application of current flow to the target region or downstream effects on functionally connected areas. Notably, only a subset of voxels identified by the seed-based RS analysis overlapped with activity elicited by either paradigm (ranging from 7.5% to 55%), with overall larger correspondence for the OLM task. Therefore, the degree of potential tDCS network effects varied substantially depending on the selected target region, the extent of its functional network and also task-specific activity patterns. (3) Finally, the degree of correspondence between imaging modalities was in part mediated by the chosen contrasts-of-interest in the task-based analyses, with more conservative control conditions resulting in reduced overlap between task-related activity and the seeded RS-network. This suggests that the choice of contrast has direct implications for revealing potential stimulation effects in tDCS-fMRI studies. In sum, the methodological approach exemplified in this study can be used for target network selection in focal Non-invasive brain stimulation (NIBS) studies and inform tDCS-fMRI data analysis and interpretation. Below, we will further discuss this novel approach and potential future extensions.

Contemporary systems neuroscience research has highlighted that higher order human brain functions rely on complex interactions between specialized brain regions, that are organized in widely distributed and partly overlapping networks (Behrens and Sporns, 2012; Breakspear, 2017). However, decisions about target selection in NIBS studies typically relied on assumptions about local processing capabilities of specific brain regions, while the origin of remote stimulation effects on brain function are currently not well understood (Meinzer et al., 2024). Notably, even some of the earliest tDCS-fMRI studies have suggested that tDCS effects may be expressed specifically within the networks of brain regions directly affected by the induced current. For example, Meinzer et al. (2012) administered conventional tDCS to left IFG during RS-fMRI and a verbal fluency task. During task-based imaging, tDCS selectively modulated activity at the stimulation site. However, in the unconstrained RS condition, tDCS increased connectivity in bilateral fronto-temporal, parietal, and premotor regions. Moreover, regions showing enhanced connectivity during RS-fMRI almost completely overlapped with two distinct networks originating from the ventral and dorsal IFG subportions, that were both directly affected by the current. These findings suggested that seed-based RS analysis may reveal areas that are potentially affected by tDCS via functional (and structural) connections and without the current reaching the connected area, thereby supporting the notion of network-based neurostimulation.

Network specific targeting is not an entirely novel approach and has recently been suggested in different contexts (Siddiqi et al., 2020; Piper et al., 2022), albeit not by using multimodal imaging. For example, a seminal study by Fox et al. (2014) employed RS-fMRI data to explore the functional connectivity between stimulation targets used in previous NIBS (cortical) and deep brain stimulation (DBS, subcortical) studies in different neuropsychiatric populations. This study confirmed that effective NIBS studies had used targets that were functionally connected to subcortical pathology foci, thereby supporting the notion of a RS network-based targeting. Likewise, we assume that neural network effects of focal tDCS are primarily expressed in brain regions that are directly connected to the target regions of NIBS, and that those can be identified by seed-based RS analysis.

This network-based perspective is also consistent with recent transcranial magnetic stimulation (TMS) studies applying the network control theory to characterize the influence of stimulation on distributed connectivity (Papallo et al., 2024; Parkes et al., 2024). For instance, Papallo et al. (2024) showed that repetitive TMS of the dorsolateral prefrontal cortex (DLPFC) alters the network-control properties of the functional connectome, with changes in these properties correlating with cognitive outcomes. In addition, an increasing number of studies are using dual-site TMS protocols such as the cortico-cortical paired associative stimulation (ccPAS), in which repeated pairs of pulses are delivered to two interconnected cortical regions at specific interstimulus intervals, aiming to modulate the strength or efficiency of the cortico-cortical pathway between them (Di Luzio et al., 2024). The majority of ccPAS studies have targeted anatomically connected pathways, particularly within motor and visual cortices, where physiological effects can be measured most directly (Hernandez-Pavon et al., 2023; Tarasi et al., 2024; Turrini et al., 2023). Such techniques can benefit from our multimodal fMRI framework for network-informed target selection, which extends beyond motor and visual cortices and potentially enhances modulation of network-level functional connectivity. Together, these findings support the view that stimulation effects can be understood in terms of a node’s capacity to shape broader network dynamics, which supports our approach to combine seed-based RS functional connectivity maps with task-evoked activity maps.

The novelty of our approach rests on the incorporation of co-acquired task-based imaging, that allowed us to determine the overlap of activity by the respective tasks with the identified seed-networks. This analysis suggested that potential tDCS effects critically depend on the interaction between the target region, its seeded network, and activity elicited by the respective experimental tasks. From a planning perspective, this approach allows to determine RS-based target networks for focal NIBS to ensure overlap with theoretically or empirically derived local nodes or networks of interest, relevant for specific tasks like OLM and APPL (e.g., Postma et al., 2008; Tagarelli et al., 2019; Abdelmotaleb et al., 2025; Kocataş et al., 2025) Moreover, our approach can also be used to determine valid active control stimulation conditions, showing limited overlap with the task-relevant networks of interest, thereby highlighting its broad applicability. However, subgroup sizes in our analyses were modest (*n* = 18 for OLM and *n* = 19 for APPL). Given the logistical and financial demands of concurrent tDCS-fMRI studies assessing both RS- and task-based paradigms, we consider the present sample sufficient for an exploratory methodological proof-of-concept study, but acknowledge that the findings should be interpreted with caution and require validation in larger cohorts.

Our analyses revealed substantial variability of correspondence between the seeded-networks and task-based activity, ranging from 7% to 55%. The overlap between RS and task-based networks was most pronounced for the OLM task, which emphasizes the potential of the OTC target region to modulate activity in a larger neural network that includes core regions of the visual-limbic network underlying spatial memory formation (Abdelmotaleb et al., 2025; Postma et al., 2008). Notably, a lower spatial overlap was observed for the APPL task (7.5%–10.6%). However, even in the complex contrast comparing APPL with a lexical decision control task (which showed the lowest overlap), a number of task-critical regions were identified, including prefrontal, premotor and insular cortices. These regions are highly relevant for language learning (Sliwinska et al., 2017; Tagarelli et al., 2019) and activity changes across learning stages predicted learning success in the original study (Kocataş et al., 2025). Hence, in specific instances, limited convergence between RS- and task-activity may be trumped by the exceptional functional relevance of the few regions identified for specific processes (i.e., the APPL task) and the lack of more substantial spatial overlap in the APPL task does not necessarily devaluate the usefulness of our approach. Indeed, relatively circumscribed overlap in task critical regions allows generating very specific, testable predictions for data analyses in tDCS-fMRI studies. Based on our results, similar variability may be expected for other task- and seed-based networks, and researchers should consider both spatial overlap and functional relevance when choosing stimulation targets.

Finally, both intrinsic and task-based networks are known to undergo changes across the human lifespan and even more so, in neuropsychiatric diseases (Greicius, 2008; Zhang et al., 2021). This needs to be taken into account in future studies planning to adopt a similar network-based targeting approach, ensuring that regions and networks of interests are derived from the target populations (older vs. young adults; patient populations vs. healthy individuals). Here, task-based fMRI can reveal changes in neural processing (e.g., to identify compensatory processes) and RS data analysis can verify that the intended target region is intrinsically connected to those regions in specific populations. This rationale is consistent with evidence suggesting that NIBS efficacy is influenced by the connectivity profiles of specific stimulation sites in specific populations, thereby enhancing its capacity to engage distal but functionally relevant regions within the targeted network (Esposito et al., 2022; Fox et al., 2012; Tan et al., 2019). Accordingly, our framework may help bridge systems neuroscience and clinical neuromodulation by supporting population specific target selection.

Regarding tDCS-related AEs, our CLMM analyses revealed no significant main effects of group or session and no group-by-session interaction on AE intensity for any symptom, indicating that AE intensity was stable across sessions and did not differ between stimulation sites. However, we found mild to moderate itching (17%) and metallic taste (4.1%) across all experimental sessions, which were within commonly reported ranges, indicating expected sensations (Antal et al., 2026). Consistent with prior work, fatigue was found to be the most frequent AE (57.1%) that often appears in sham and active tDCS conditions (Brunoni et al., 2011; Delicado-Miralles et al., 2024), likely reflecting procedural burden and expectations rather than direct effects of stimulation alone. Burning sensation was also frequently reported; the total incidence of burning (38.8%) aligns with a recent study by El Jamal et al. (2023), who reported burning in 40.5% and 32.6% of sham sessions in tDCS-naïve participants and those who experienced multiple sessions, respectively. Notably, more pronounced burning sensations were more frequently observed during sham compared to active stimulation in tDCS-naïve participants (El Jamal et al., 2023). Despite some discomfort, all participants well tolerated the focal tDCS sessions and completed the experiment, in line with recent findings from similarly complex tDCS protocols, which also reported high tolerability and comparable AE profiles in both active and sham conditions at intensities up to 4 mA (El Jamal et al., 2023; Reckow et al., 2018).

In sum, future studies are indicated to further validate this framework in larger and independent samples and extend it to additional tasks, target regions, and populations. In particular, the approach should be tested in older adults and clinical groups, where intrinsic and task-based networks may differ from those observed in healthy young adults. Future work could also incorporate individualized current modeling (Niemann et al., 2024a) and more realistic seed regions, representing areas where focal tDCS induces a physiologically relevant current dose. This would allow a more precise estimate of the networks directly affected by NIBS and further improve the interpretation of stimulation effects in tDCS-fMRI studies.

Aside from planning future tDCS studies, our approach also allows constraining data analyses in tDCS-fMRI studies to brain regions where neural network effects of NIBS are to be expected, thereby providing an empirically informed approach for hypothesis testing. Unlike task-based imaging, RS-fMRI data is readily available from publicly available databases, including lifespan data to account for neural network reorganization due to aging (e.g., www.humanconnectome.org). This makes our framework attractive for prospective network-based targeting approaches and data analysis, with potential to rule out alternative explanations for behavioral or neural modulation like current spread to unintended target regions (Ito et al., 2017). Nonetheless, complex cognitive tasks (like OLM or APPL) typically involve several spatially or temporally distinct networks and effective processing relies on the delicate and time varying balance between them (Ren et al., 2017; Williams et al., 2022; Zhang et al., 2023). Such potential effects also need to be taken into account when planning and analyzing future tDCS-fMRI studies.

## 5 Conclusion

This study establishes a principled, cross-modal fMRI framework that bridges a critical gap in neuromodulation research. By integrating reliable intrinsic connectivity maps with task-evoked activity patterns, it provides a method to prospectively identify network-level targets for focal NIBS and to generate hypotheses for data analysis in tDCS-fMRI studies. This approach provides a foundation for future studies investigating the modulation of specific functional networks, with potential to facilitate a shift from stimulating single brain regions to targeting predefined functional pathways. However, larger-sample follow-up studies are needed to consolidate these initial findings.

## Data Availability

The study was pre-registered with the Open Science Framework (OSF), and the protocol can be accessed through OSF Registries (https://osf.io/t37u2). To ensure full transparency and reproducibility, our analysis scripts are publicly available on GitHub (https://github.com/ShahAliR/NetworkOverlap-Task-RS-fMRI). Due to ethical and privacy concerns about the study’s participants, the row data are not publicly accessible. However, the corresponding author can provide the data supporting the study’s conclusions upon request.

## Footnotes

1
https://www.memoslap.de/en/research/
